# Impact of an oral care subsidization reform on intersectional inequities in self-rated oral health in Sweden

**DOI:** 10.1186/s12939-024-02121-7

**Published:** 2024-03-19

**Authors:** Cynthia Anticona, Anna Liisa Suominen, Pernilla Lif Holgerson, Per E Gustafsson

**Affiliations:** 1 Department of Epidemiology and Global Health, Umeå University, Umeå, SE-90187 Sweden; 2 Department of Odontology, Umeå University, Umeå, SE-90187 Sweden; 3 Institute of Dentistry, University of Eastern Finland, Kuopio, Finland; 4 Oral and Maxillofacial Teaching Unit, Kuopio University Hospital, Kuopio, Finland; 5 Department of Odontology, Section of Pediatric Dentistry, Umeå University, Umeå, SE-90187 Sweden

**Keywords:** Oral health care, Social inequities, Self-rated oral health, Subsidization reform, Intersectionality, Sweden

## Abstract

**Background:**

Oral health in Sweden is good at the population level, but seemingly with persisting or increasing inequities over the last decades. In 2008, a major Swedish reform introduced universal partial subsidies to promote preventive care and reduce the treatment cost for patients with extensive care needs. This study aimed to apply an intersectional approach to assess the impact of the 2008 subsidization reform on inequities in self-rated oral health among adults in Sweden over the period 2004–2018.

**Methods:**

Data from 14 national surveys conducted over 2004–2018 were divided into three study periods: pre-reform (2004–2007), early post-reform (2008–2012) and late post-reform (2013–2018). The final study population was 118,650 individuals aged 24–84 years. Inequities in self-rated oral health were examined by intersectional analysis of individual heterogeneity and discriminatory accuracy across 48 intersectional strata defined by gender, age, educational level, income, and immigrant status.

**Results:**

Overall, the prevalence of poor self-rated oral health decreased gradually after the reform. Gender-, education- and income-related inequities increased after the reform, but no discernible change was seen for age- or immigration-related inequities. The majority of intersectional strata experienced patterns of persistently or delayed increased inequities following the reform.

**Conclusions:**

Increased inequities in self-rated oral health were found in most intersectional strata following the reform, despite the seemingly positive oral health trends at the population level. Applying an intersectional approach might be particularly relevant for welfare states with overall good oral health outcomes but unsuccessful efforts to reduce inequities.

**Supplementary Information:**

The online version contains supplementary material available at 10.1186/s12939-024-02121-7.

## Background

An overall improvement in oral health has occurred in most high- and middle-income countries over recent decades, but this has not equally benefited all members of society [1, 2]. Inequities in oral health refer to unfair and avoidable differences between social groups, with a worsened status at each point as one descends in the social hierarchy [1]. While tackling inequities requires multisectoral concurrent actions by international organizations, governments, health systems, health workforce, and communities, the role of the health system is particularly important to provide equal access to good quality care. Furthermore, a focus on population coverage of preventive care is regarded as a key strategy to achieve equity, as it is economically sustainable, socially desirable and ethically responsible [3, 4]. However, effective equity promotion requires preventive care to be carefully designed with a clear inclusive approach, as otherwise inequities can increase, leading to a greater demand for curative care to counteract the inequities [3]. Moreover, policies and interventions should primarily focus on addressing the social determinants of oral health, taking into account the extent to which they vary within, between, and among groups, to tailor the needs of each group [1, 3]. Nevertheless, research that examines policies and interventions with a comprehensive and nuanced equity lens is scarce in most countries [1, 3].

In Sweden, overall oral health is regarded as good from an international perspective [2, 5]. However, evidence from national reports and a few cross-sectional studies over the last couple of decades has shown persisting and even increasing oral health inequities [5–9], with particular groups, including individuals with low educational level, low income and immigrants, left behind [5]. This picture does not reflect the ambitions of equitable oral health for the whole population as stipulated in the Swedish Oral Care Act [5] nor the eradication of health inequities, stated as an overarching goal in national health policy [10].

The approach of the Swedish oral care system to achieve equitable oral health has to date focused on several financial policy instruments. These measures are considered generous by European standards but remain considerably weaker than the subsidization of the predominantly publicly funded and provided general healthcare [2]. Since the early 1970s, the organization of the Swedish oral care system has included free oral care for children and youth and partial subsidies for the adult population. The partial subsidies, however, have been subject to several changes over time. In 1974, an oral care insurance was introduced as part of the general health insurance, covering up to 75% of the treatment costs, with costs regulated for both public and private caregivers. Due to the large financial costs of the insurance, it was significantly reduced in 1999. The 1999 insurance provided: (i) a compensation of 30% of basic oral care only (considered as procedures necessary for achieving a functionally and visually acceptable oral status e.g., oral fillings and extractions) but which, to limit overall costs, excluded regular oral examinations for all adults above 29 years; and (ii) a high-cost protection for “non-basic care” procedures, excluding fixed prosthetics and orthodontics. At the same time, oral care prices became deregulated [5]. Further evaluations of the 1999 insurance suggested the need for a new reform to achieve the Swedish Oral Care Act´s aim of equal oral health and care for all [5]. Thus, in 2008, a major reform introduced universal partial subsidies that aimed to promote preventive care with emphasis on regular oral examinations. In addition, the existing high-cost protection subsidy was strengthened to offer care at reasonable costs for those with extensive treatment needs. The scheme consisted of a small fixed annual sum of 150 SEK (13 EUR in 2024) to all adults, with double the amount for the younger (24–29 years) or the older groups (≥ 75 years) and a high-cost protection subsidy of 50% above 3000 SEK (260 EUR) and 85% above 15,000 SEK (1300 EUR). While the high-cost protection has remained unchanged since its introduction in 2008, the subsidy system was strengthened by doubling the amount for all groups in 2018 [5].

Although the ultimate goal of the 2008 reform was to advance equity in oral care and health, this has not been scientifically evaluated. Governmental reports have only shown a limited increase in oral services utilization and failure of the high-cost protection to benefit groups with low income and extensive oral care needs [11].

This scarcity in scientific evidence is particularly noticeable when compared to the available literature on oral care subsidization reforms in the neighboring country of Finland. Similar to Sweden and the other Nordic countries, Finland shares a welfare state model aimed to provide equal care access, with a prominent role of the public sector in adult oral care [5].

Research on the Finnish universal coverage of subsidized oral care implemented in 2001–2002 showed an overall increase in oral services utilization and a decrease both in the perceived need for oral care and perceived poor oral health in a 5-year follow-up [12–14]. Nevertheless, a small reduction in inequities both in oral services utilization and oral health outcomes (e.g., self-rated oral health) detected in a 3-year follow-up was only temporary, as inequities turned to the same levels or even widened in a 5-year follow-up [13, 14].

Building upon these studies, further research would benefit from using a holistic approach that accounts for the complex picture of oral health inequities, with heterogenic patterns of social gradients across all inequity dimensions. In this regard, a novel approach to equity, guided by the concept of intersectionality, has recently gained interest within the field of oral health [4, 15]. Intersectionality theory recognizes that individuals´ lives are influenced by the simultaneous interaction of multiple dimensions of inequities [15, 16]. Accordingly, empirical research explores intersectional strata defined by combinations of several inequity dimensions, providing a more comprehensive and nuanced mapping of social inequities compared to examining one dimension at the time [16, 17].

Despite the increasing interest in intersectionality, a limited number of empirical health studies globally have used this approach, and only a few have focused on oral health and care outcomes [18, 19]. In a recent study, we were able to identify unforeseen inequities in oral care in Sweden, e.g., certain groups paradoxically remaining at high risk for unmet oral care needs despite having high income [19].

The present study aims to apply an intersectionality perspective to ascertain whether the currently valid oral care subsidization reform, implemented in Sweden in 2008, helped decrease inequities in self-rated oral health among adults, in the period 2004–2018.

## Methods

### Design and ethics

Data came from the complete series of the survey Health on Equal Terms (HET) conducted annually by the Public Health Agency of Sweden in the period 2004–2018, except for 2017 when no survey was conducted. Data including 2018 were considered, as the strengthening of subsidies in 2018 was implemented during the months of the survey roll-out, which was deemed too short time to potentially impact oral health inequities and thus bias the assessment of the 2008 reform. Data were divided into three study periods: pre-reform = 2004–2007; early post-reform = 2008–2012 and late post-reform = 2013–2018. These cut-off points were determined based on previous studies on the 2001–2002 Finnish reform, reporting initial small reductions in oral care inequities three years after the reform, but which after five years had turned to higher levels of inequities than before the reform [13, 14].

The HET survey gathers information about general and oral health and care utilization in the Swedish population aged 16 to 84 years. The survey response rate displayed a decreasing trend over the years (from 60.8% in 2004 to 42.1% in 2018) resulting in a total of 136,301 responses with an overall response rate of 50.5%. Data from respondents aged 16–23 years [*N* = 13,647 (10.0%)] were excluded as the reform did not target this group. Thereafter, respondents with missing responses on the outcome and exposure variables were excluded [*N* = 4,004 (3.3%)]. The final study population was *N* = 118,650 (87% of all respondents).

This study was approved by the Swedish Ethical Review Authority (approval no. 2021–02398).

### Variables

The outcome variable, *self-rated oral health* (SROH), is considered a comprehensive, composite indicator grounded in holistic models that conceptualize health beyond disease and impairment [20]. Apart from reflecting disease, it is informed by functional capacity, pain, aesthetics, psychological and psychosocial elements [21]. Thus, it is well-used in public health surveys as a valid, reliable and cost-effective way of capturing SROH [22]. The survey question to assess this variable was: “How is your oral health?” (Hur tycker du att din tandhälsa är?), and the response options were ‘very good’, ‘good’, ‘more less’, ‘poor’, and ‘very poor’. These were dichotomized based on the Public Health Agency of Sweden classification into good SROH = 0 (‘very good’, ‘good’) and poor SROH = 1 (‘more less’, ‘poor’, ‘very poor’) [23]. Note that although the Swedish question directly translates to “dental health”, it has a broader meaning than only teeth status. We have therefore used “oral health” to refer to the outcome, as has been done by previous Swedish studies [8, 9].

The exposure variables consisted of five sociodemographic inequity dimensions, which were retrieved and linked from population registers of Statistics Sweden. They were categorized and coded as follows: gender, defined by the proxy variable of biological sex (woman = 1; man = 0); age (24–44 years = 2; 45–64 years = 1 and 65–84 years = 0), educational level (low = 1[< 3 years of high school]; high = 0 [≥ 3 years of high school]), immigrant status determined at any point in life having immigrated to Sweden (immigrant = 1; native = 0), and individual disposable income (low = 1[< median]; high = 0[≥ median]) adjusted for inflation, using official consumer price indices.

All the categories were subsequently cross classified to create a multicategorical variable comprising 48 mutually exclusive intersectional strata or complex social positions. The reference stratum were men, aged ≥ 65 years, native, with high education and high income.

### Analysis

An intersectionality-informed analysis of individual heterogeneity and discriminatory accuracy (AIHDA) was conducted based on the procedure described elsewhere [17]. In the individual heterogeneity component, the outcome is modelled using a regression analysis of individuals nested within a matrix defined by the intersection of several inequity dimensions (intersectional strata). Subsequently, the discriminatory accuracy component provides information on the accuracy that the inequity dimensions in the model discriminate individuals who have the outcome from those who do not [16].

First, prevalence ratios (PR) with 95% confidence intervals (CI) of poor SROH were estimated by generalized linear models using log family and identity link, separately for the pre-reform, early post-reform, and late post-reform periods in two consecutive models. Model 1 consisted of a base model including the five single inequity dimensions, while model 2 used the intersectional strata variable instead of the single dimensions. The PR differences between the study periods (period 2 versus [vs] 1, period 3 vs. 2 and period 3 vs. 1) were examined using interaction analysis with group * period interaction terms. One intersectional stratum (immigrant older women with high income and low education) only contained two observations for the pre-reform period, and its prevalence was therefore not possible to estimate.

Second, the discriminatory accuracy of the single inequity dimensions and the intersectional strata was estimated by calculating the area under the receiver operating characteristic curve (AU-ROC, or AUC for short) for the corresponding six models. A previously proposed classification was used to interpret the discriminatory accuracy as follows: (i) ‘absent or very small’ (AUC = 0.5–0.6), (ii) ‘moderate’ (AUC > 0.6-≤0.7), (iii) ‘large’ (AUC > 0.7-≤0.8) and (iv) ‘very large’ (AUC > 0.8) [16]. The incremental change in the AUC value (ΔAUC) was calculated to quantify the improvement in the discriminatory accuracy between models 1 and 2. The differences in AUC between the different periods were also estimated to estimate any change in discriminatory accuracy following the reform. All analyses were performed in STATA 14.0.

## Results

### Characteristics of the study population

The size of the study population increased over the three consecutive periods, with 20%, 37% and 50% of the population in the pre-reform, early post-reform, and late post-reform periods, respectively. However, the relative distribution of most of the inequity dimensions was similar in the three periods, with the majority being women, aged 45–64 years, native and highly educated. Income was the exception, as the majority of participants in the first two periods had low income, while the majority in the third period had high income (Table 1).

**Table 1:** Distribution and prevalence of poor self-rated oral health (SROH) across inequity dimensions and study periods

	Overall study period 2004–2018		Pre-reform 2004–2007		Early post-reform 2008–2012		Late post-reform 2013–2018	
	Total	Poor SROH	Total	Poor SROH	Total	Poor SROH	Total	Poor SROH
Total sample	118,650 (100)	30,296 (25.53)	24,263 (100)	6,686 (27.56)	44,148 (100)	11,565 (26.2)	50,239 (100)	12,045 (23.98)
Inequity dimensions								
Gender								
Man	54,190 (45.67)	15,329 (28.29)	11,036 (45.48)	3,284 (29.76)	19,938 (45.17)	5,820 (29.19)	23,216 (46.21)	6,225 (26.81)
Woman	64,460 (54.32)	14,967 (23.22)	13,227 (54.51)	3,402 (25.72)	24,210 (54.82)	5,745 (23.73)	27,023 (53.78)	5,820 (21.54)
Education								
High	68,407 (57.65)	14,944 (21.85)	12,803 (52.76)	3,020 (23.59)	24,983 (56.58)	5,666 (22.68)	30,621 (60.95)	6,258 (20.44)
Low	50,243 (42.34)	15, 352 (30.56)	11,460 (47.23)	3,666 (31.99)	19,165 (43.41)	5,899 (30.78)	19,618 (39.04)	5,787 (29.50)
Age (years)								
65–84	33,550 (28.27)	8,972 (26.74)	4,190 (17.26)	1,268 (30.26)	11,901 (26.95)	3,298 (27.71)	17,459 (34.75)	4,406 (25.24)
45–64	47,802 (40.28)	12,172 (25.46)	10,539 (43.43)	2,895 (27.47)	18,168 (41.15)	4,800 (26.42)	19,095 (38.00)	4,447 (23.45)
24–44	37,298 (31.43)	9,152 (24.54)	9,534 (39.29)	2,523 (26.46)	14,079 (31.89)	3,467 (24.63)	13,685 (27.23)	3,162 (23.11)
Income								
High	57,714 (48.64)	11,998 (20.79)	8,617 (35.51)	1,876 (21.77)	20,606 (46.67)	4,502 (21.85)	28,491 (56.71)	5,620 (19.73)
Low	60,936 (51.35)	18,298 (30.03)	15,646 (64.48)	4,810 (30.74)	23,542 (53.32)	7,063 (30.00)	21,748 (43.28)	6,425 (29.54)
Immigration								
Native	104,204 (87.82)	35,396 (24.37)	22,968 (94.66)	6,195 (26.97)	37,585 (85.13)	9,213 (24.51)	43,651 (86.88)	9,988 (22.88)
Immigrant	14,446 (12.17)	4,900 (33.92)	1,295 (5.33)	491 (37.92)	6,563 (14.86)	2,352 (35.84)	6,588 (13.11)	2,057 (31.22)

Numbers are N (%)

The prevalence of poor SROH showed a slight decrease over time (27.5%, 26.2% and 23.9% in the pre-reform, early post-reform, and late post-reform period respectively). Additionally, SROH showed a similar social patterning constant throughout the study period, with men, participants aged ≥ 65 years, immigrants, and those having low education or low income reporting a higher prevalence of poor SROH (Table 1).

### Inequities in SROH by single dimensions

Table 2 shows the relative inequities in SROH [prevalence ratio (PR)] by single inequity dimensions in the three study periods, considering both the main effects and the interaction of the three periods. Changes in the magnitude of oral health inequities over time varied according to inequity dimension and study period, with overall increasing inequities by gender, education and income, but no discernible change for age- or immigration-related inequities. Specifically, the gender gap increased early on, from 14% lower risk among women (pre-reform) to 19% (early post-reform) and 20% (late post-reform) (PR_interaction_ = 0.92; 95% CI = 0.88–0.97). Education- and income-related inequities instead increased significantly only during the late post-reform period, from 35% (pre-reform) and 36% (early post-reform) higher risk among low-educated groups, rising to 44% in the late post-reform period (PR_interaction_ = 1.06; 95% CI = 1.01–1.12), and from 41% (pre-reform) and 37% (early post-reform) higher risk among low-income groups, increasing to 49% in the late post-reform period (PR_interaction_ = 1.09; 95% CI = 1.00-1.12).

**Table 2:** Poor self-rated oral health by inequity dimension in the study periods (main and interaction effects)

	Main effects			Interaction effects		
	Pre-reform	Early post-reform	Late post-reform	Early post- vs. pre-reform	Late post- vs. early post-reform	Late post reform vs. pre-reform
Inequity dimensions						
Gender						
Man	1	1	1	1	1	1
Woman	0.86 (0.82–0.90)	0.81 (0.79–0.84)	0.80 (0.77–0.82)	0.94 (0.98–0.99)	0.98 (0.94–1.03)	0.92 (0.88–0.97)
Education						
High	1	1	1	1	1	1
Low	1.35 (1.30–1.41)	1.36 (1.32–1.40)	1.44 (1.39–1.48)	1.00 (0.95–1.05)	1.06 (1.01–1.11)	1.06 (1.01–1.12)
Age (years)						
65–84	1	1	1	1	1	1
45–64	0.90 (0.86–0.96)	0.95 (0.91–0.99)	0.92 (0.89–0.96)	1.05 (0.98-1-12)	0.97 (0.92–1.02)	1.02 (0.95–1.09)
24–44	0.87 (0.82–0.92)	0.88 (0.85–0.93)	0.91 (0.87–0.95)	1.01 (0.94–1.08)	1.03 (0.97–1.09)	1.04 (0.97–1.12)
Income						
High	1	1	1	1	1	1
Low	1.41 (1.34–1.47)	1.37 (1.32–1.41)	1.49 (1.45–1.55)	0.97 (0.91–1.02)	1.09 (1.04–1.14)	1.06 (1.0-1.12)
Immigrant status						
Native	1	1	1	1	1	1
Immigrant	1.41 (1.31–1.51)	1.46 (1.40–1.51)	1.36 (1.31-1-41)	1.04 (0.95–1.12)	0.93 (0.88–0.98)	0.97 (0.89–1.05)

Numbers are prevalence ratios with 95% confidence intervals

### Intersectional inequities in SROH

**Fig. 1:**
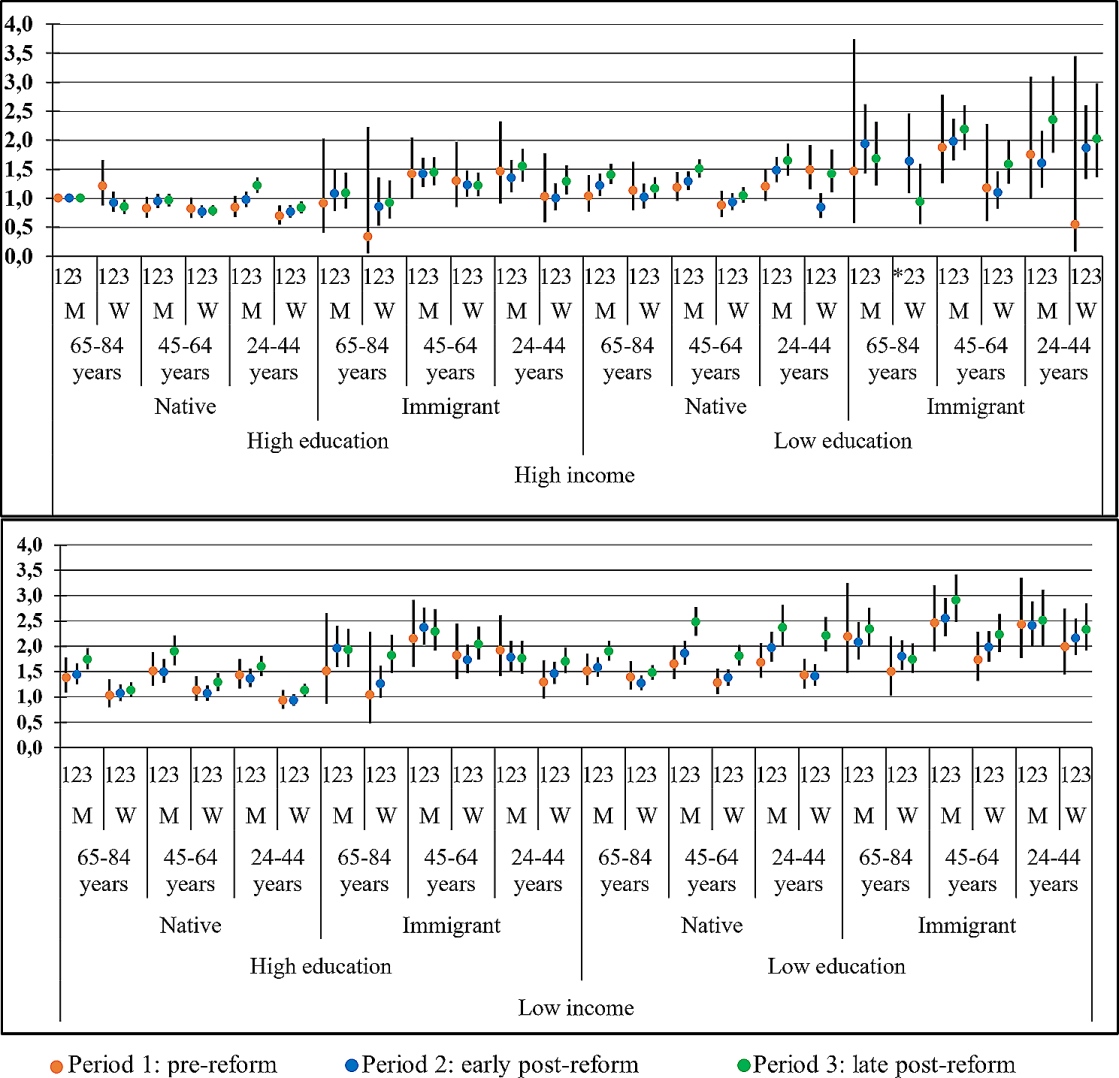
Relative inequities of poor self-rated oral health for 48 intersectional strata in the study periods. Estimates are prevalence ratios and 95% Confidence intervals, with native men aged ≥ 65 years with high education and high income as the reference stratum. *Missing estimate. M = man; W = woman

The prevalence of SROH across intersectional strata (reported in full in Supplementary Table S1) displayed a large variation in all three periods, ranging from 7.7 to 55.6% in the pre-reform; 16.3–54.2% in the early post-reform and 14.1–52.1% in the late post-reform period. The intersectional inequities, illustrated by the stratum-specific prevalence ratios (95% CI) in Fig. 1, displayed both general and specific patterns. In all three study periods, the stratum with the highest risk for poor SROH comprised the group of immigrant men aged 45–64 years with low-income and education (PR = 2.46, 2.54, and 2.90 in the pre-, early post-, and late post-reform period, respectively). Indeed, the five strata with highest risk (PR = > 2.40) included men younger than 65 years who had low education and income. In contrast, similarly aged (45–64 years) immigrant men, but instead with high education and income, reported a relatively low risk (PR = ≤ 1.30) in the three periods. In the other end of the spectrum, the strata with lowest risk (PR = < 0.80) all included women of any age who had high education and income (Fig. 1). A notably high risk, double that of the reference group (PR = 2.20) was, however, found among Swedish-born young women but with low education and income.

**Fig. 2:**
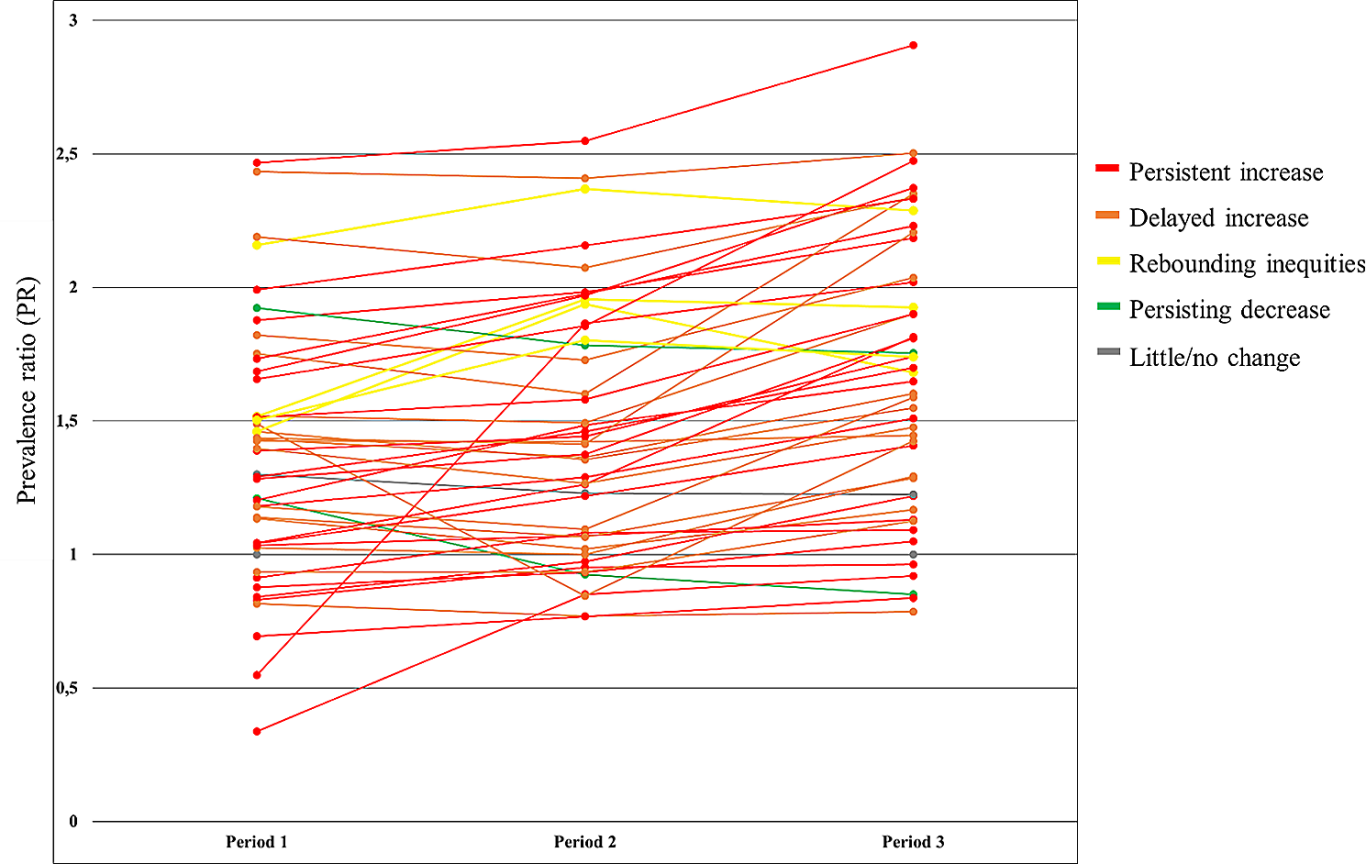
Change in relative inequities of poor self-rated oral health for 48 intersectional strata. Points represent prevalence ratios of poor self-rated oral health, with native men aged ≥ 65 years with high education and high income as the reference stratum

An overview of the changes in stratum-level intersectional inequities in SROH across the three study periods is illustrated descriptively in Fig. 2 (and reported in full in Supplementary Table S1). Overall, inequities tended to increase from the pre-reform to the late post-reform period, but the increases were more commonly attributed to the late rather than early post-reform period. Specifically, the majority of the 47 non-reference intersectional strata displayed either patterns of *persistent increase* in inequities across the three study periods (percentual change in PR > 3.31% for P2 vs. P1, > 1.08% for P3 vs. P2; k = 22 strata) or a *delayed increase* illustrated by an initial decrease in inequities followed by an increase in the late post-reform period (<-0.09% for P2 vs. P1, > 1.70% for P3 vs. P2; k = 17 strata). A minority of strata displayed patterns of *persisting decreased inequities* (<-7.26% for P2 vs. P1, <-1.64% for P3 vs. P2; k = 2 strata), *rebounding* inequities with early increase followed by decreasing inequities in late post-reform period (> 9.75% for P2 vs. P1, <-1.57% for P3 vs. P2; k = 4 strata), or of little or *no change* (-5% for P2 vs. P1 and − 0.4% for P3 vs. P2; k = 1 stratum). The small sample size of the remaining stratum allowed the comparison of only the second and third periods, showing increased inequities (not shown in Fig. 2). The largest increase in inequities from the pre- to the late post-reform period (> 70%) was found in three strata, all including immigrant women. Conversely, the largest decrease (~ -30%) was found in one stratum including native women aged 65–84 years with both high education and income.

Due to the low strata-level sample size, the comparisons between periods were only significant for 12 strata, almost all (*n* = 11) including the larger-sized strata of native participants. Of these, eight strata displayed significantly increased inequities from the pre-reform to the late post-reform period, and in four of these significantly increased inequities were found from the pre-reform to the early post-reform period (Supplementary Table S1).

### Discriminatory accuracy

The discriminatory accuracy for model 1 was low for the pre-reform period (AUC = 0.59) and moderate for the early post-reform (AUC = 0.60) and late post-reform period (AUC = 0.61). Incorporating the intersectional strata in model 2 led to a slight improvement in discriminatory accuracy in all periods (ΔAUC pre-reform = 0.01, ΔAUC early post- and late post-reform = 0.004). The discriminatory accuracy improved slightly but significantly across the study periods for both single dimension and intersectional strata models (Table 3).

**Table 3:** Discriminatory accuracy of the intersectional model in the study periods

	Model estimates			Model comparisons		
	Pre-reform	Early post-reform	Late post-reform	Early post- vs. pre-reform	Late post- vs. early post-reform	Late post-reform vs. pre-reform
Model 1 single dimensions	0.59 (0.58–0.60)	0.61(0.59–0.61)	0.62 (0.61–0.62)	*p* < 0.05	*p* < 0.05	*p* < 0.01
Model 2 intersectional strata	0.60 (0.59–0.61)	0.61(0.60–0.61)	0.62 (0.61–0.62)	*p* < 0.05	*p* < 0.01	*p* < 0.01
ΔAUC*	0.01	0.004	0.004	0.01	0.004	0.004

Estimates are reported as Area Under the Receiver Operating Characteristic Curve (AUC) and 95% confidence interval

*model comparison refers to Model 2

## Discussion

This study examined inequities in SROH in the Swedish adult population before and after the 2008 subsidization reform across gender, age, immigration, education and income. Overall, a gradually decreasing prevalence of poor SROH was seen after the reform, but complex patterns of increasing inequities were simultaneously observed. The intersectional analysis showed increasing inequities in the majority of groups, including several groups of young adults and immigrants for whom inequities were invisible in single-dimension analyses.

The overall improvement in SROH observed over the study period likely corresponds to the steady positive trends in oral health in Sweden reported since 2004, which has been attributed to the enhancement of preventive and curative care strategies by the Swedish health system [5]. Such strategies, however, tend to disproportionally benefit the most socially advantaged groups (who are more likely to use care services), and subsequently widen inequities [3], as also was observed in the present study. The perception of poor SROH displayed a social patterning that remained after the reform, being more frequent among men, immigrants, those with low education or low income. This pattern is consistent with previous research, suggesting that socially disadvantaged people have the worst perception of their oral health [21]. Although there is less consensus regarding the most disadvantaged age group, our findings are similar to other studies arguing that the elderly’s perception of oral health might be particularly affected by common age-related health problems, e.g., comorbidity of systemic diseases, disability and tooth loss [9, 24].

In contrast to the social patterning, the magnitude of inequities in SROH varied by inequity dimension and time since the introduction of the reform. Gender inequities (higher risk among men) increased promptly after the reform and persisted at a higher level, while education and income-related inequities only increased later, after several years into the reform, and with little change seen for age and immigration-related inequities. Relatedly, research assessing inequities in self-rated oral health after the Finnish reform reported that increased inequities were mainly explained by health, oral care and socioeconomic factors, particularly income [14].

The overall patterns described above were further disentangled by the intersectional examination. First, the discriminatory accuracy of individual inequity dimension and of intersectional strata was higher in the post-reform period, which suggests generally increased inequities following the reform. Moreover, in the mapping of inequalities, two intersectionality-related hypotheses were visualized in all periods. These include the synergistic effect of the multiple social disadvantages on the risk for poor SROH and the contingency of inequities, postulating that the risk of a particular social location is modified by the social position along other inequity dimensions. For example, being an immigrant did not imply a higher risk of poor SROH for old women with both high education and income but did matter for younger women (45–64 years), despite their high education and income. Second, after the introduction of the reform, most intersectional groups experienced early increased inequities that persisted, followed by a smaller group for whom inequities increased only in the late follow-up period. Finally, a minority of groups showed either persistently decreased inequities, little change or rebounding inequities. Examples of early and late increased inequities were seen in most groups of young adults, but with the greatest gap among those with low income. It is possible that young adults’ perception of oral health worsens when they are no longer considered a responsibility of the public system and need to seek and pay for oral care themselves. Although no change was detected for immigration-related inequities in the single-dimension analysis, persistently increasing inequities were seen in the groups of immigrant men of any age with both low income and education, as well as in the corresponding group of native men.

Taken together, these findings suggest that the reform did not advance equity in oral health, which would be expected to result from a more equitable oral services utilization following the subsidies. Certainly, reports indicate a marginal overall increase in the frequency of oral visits (2% between 2009 and 2011) and failure of the high-cost protection to reach all who had large care needs [11]. One explanation might be that the subsidies did not represent a real incentive to seek oral care for all social groups. In 2009, the subsidy covered only 20% of the cost of an oral examination for most adults and the double for the youngest (24–29 years) and oldest groups (> 64 years) [11]. Additionally, the out-of-pocket amount to qualify for the high-cost protection might have been unaffordable for low-income groups [11]. However, the more extensive coverage provided by the Finnish reform did not either contribute to reduce oral health inequities [12]. In a 5-year follow-up of the Finnish reform, no change was noted in either the prevalence of poor perceived oral health or the level of income-related inequality in perceived oral health. Moreover, although the overall increase in oral services utilization in Finland might have been higher than that in Sweden (6% between 2001 and 2007 vs. 2% between 2009 and 2011), this could be regarded as modest in relation to the larger magnitude of the subsidies [12, 14]. It might be argued that common factors affecting the oral services utilization and increased inequities in both countries included the restrictions for subsidized prosthetic treatments and the deregulated prices for oral care. Thus, specific services that are commonly needed among the older population were excluded from the benefit programs which likely increased age-related inequities. Additionally, large disparities in the price of care between the private and public sector might affect the quality of service and subsequently, the perception of health. Finnish adults receiving private oral care were more likely to be healthier and to have high income than those attending public clinics after the reform [12].

Nevertheless, it has been recognized that reducing financial barriers to access oral care might not be sufficient to tackle social inequities in oral health [3, 14, 24] and other accessibility-related dimensions including the availability of care services and the willingness and ability to seek care should be addressed. Furthermore, upstream actions focused on social determinants of oral health beyond the health system have been widely advocated to tackle oral health inequities. That means policy/interventions that create structural conditions for people to make good choices regarding the main proximal factors of oral health/disease, e.g., the legal right to time off work for a dental appointment (upstream action) can encourage the preventive care-seeking behavior (downstream action) [3, 25]. Similar arguments have been noted in other contexts e.g. South Korea [26] and Brazil [27]. The income gradient in self-reported oral health among adults in South Korea remained the same eight years after the expansion of a universal health insurance to include oral health services in 2009 [26]. Meanwhile, in Brazil, high levels of inequity in access to oral services were maintained ten years after the implementation of universal coverage of free public oral care in 2003 [27].

Our findings suggest that the subsidization reform of 2008 was ineffective to reduce inequities in SROH. However, further research is needed to investigate the reasons, e.g., whether the subsidies were too small to have any meaningful effect countering the inequities, or if financial subsidies alone are insufficient because they fail to address other determinants of oral care inequities, e.g., barriers for the physical access to oral care experienced by people living in rural areas or individuals with severe illnesses/disabilities.

Relatedly, previous research suggests that universal measures of financial protection (e.g. partial subsidies) might not be sufficient to reduce oral health inequities, and could even induce inequities if the benefit packages have a limited scope in the coverage of treatment costs, and in the presence of a strong private sector that creates differences in the service quality between public and private providers [28]. Financial mechanisms targeting exclusively the most deprived individuals would not be either a solution because the social gradient affects the whole population. In this respect, the proportionate universalism approach has been advocated as effective to tackle income inequities, as universal actions are implemented at a scale and intensity that is proportionate to the level of need and/or disadvantage [29]. Further research evaluating programs based upon proportional universalism would be therefore beneficial for future policy planning.

This study assessed inequities in SROH as a potential distal outcome of the intended increase in oral services utilization. SROH is regarded as a dynamic feature associated with clinical oral status as well as several other psychological, cultural and social factors that are unlikely to be affected by a specific financial reform [14]. Further research assessing inequities in oral services utilization would be needed to clarify whether SROH inequities can be explained by inequities in utilization, data that were not available in the HET survey. Another limitation relates to the declined response rates during the study period and the underrepresentation of certain intersectional groups, particularly immigrants. However, there was a similar distribution of the inequity dimensions during the study period, making the results comparable across all three periods. Finally, the repeated cross-sectional design of this study did not allow us to make causal inferences, and the small number of time points before and after the reform might have confounded a real effect of the reform from general time trends in SROH inequities.

## Conclusions

In summary, the findings reveal a close-to universality of increased inequities in SROH following the reform across complex social positions, despite the seemingly positive oral health trends at the population level. This illustrates that the intersectional approach enables the identification of vulnerable groups, which can remain undiscernible when analysing dimensions as separate entities. The application of this approach might be particularly relevant for welfare states such as the Nordic countries, where the population displays good overall oral health, but corresponding inequities seem entrenched and particularly difficult to address. Even if the observed increased inequities cannot be confidently attributed to an effect of the reform, the reform at the very least has been insufficient in counteracting the rising oral health inequities in Sweden. Indeed, the challenges for equitable oral care in Sweden have been recently brought to the forefront of multiple stakeholders’ agenda, and future reforms of the Swedish oral health system are expected. In this context, further research is needed to identify the reasons for the apparent failure of the studied reform to promote equity in a variety of outcomes as well as more generally to ascertain the effects of oral care reforms on different outcomes, e.g., self-reported oral health measures, registries of oral services utilization and clinical parameters, and across relevant inequity dimensions.

### Electronic supplementary material

Below is the link to the electronic supplementary material.

Supplementary Material 1

## Data Availability

The study used secondary data protected by strict secrecy, as regulated by the Public Access to Information and Secrecy Act (2009:400), which, however, contains an exception for research. After formal application to the responsible authority, The Public Health Agency of Sweden, and contingent on vetting by the Swedish Ethical Review Authority, access can therefore be granted to personal data for research purposes.

## Abbreviations

SROH: Self-rated oral health
SEK: Swedish Crown
EUR: Euro
HET: Health at Equal Terms
AIHDA: Analysis of individual heterogeneity and discriminatory accuracy
vs: versus
PR: prevalence ratio
CI: Confidence interval
AUC: Area Under the Curve
