# Supplementary material for: Impact of an oral care subsidization reform on intersectional inequities in self-rated oral health in Sweden
Source: Int J Equity Health. 2024 Mar 19;23:63. doi: 10.1186/s12939-024-02121-7 (PMC10953229; doi:10.1186/s12939-024-02121-7)
Supplement: Supplementary file 1 — Supplementary Material 1 [file 12939_2024_2121_MOESM1_ESM.pdf]

Supplementary Table S1. Distribution, prevalence, prevalence ratios (PR) with 95% CI of poor self-rated oral health (SROH) and inequities ‘change over pre-reform (P1), early post-reform (P2) and late post-reform (P3) period in 48 intersectional strata.

Intersectional stratum	Pre-reform 2004-2007			Early post-reform 2008-2012			Late post-reform 2013-2018			P2 vs P1 (%)	P3 vs P2 (%)	P3 vs P1 (%)	Overall change
	Total N (%)	Poor SROH N (%)	PR (95% CI)	Total N (%)	Poor SROH N (%)	PR (95% CI)	Total N (%)	Poor SROH N (%)	PR (95% CI)				
Total	24,263 (100)	6,686 (27.56)		44,148 (100)	11,565 (26.2)		50,239 (100)	12,045 (23.98)					
HiInc, HiEd, Swe, 65-84 years, M	381 (1.57)	87 (22.83)	Ref	1,289 (2.91)	274 (21.26)	Ref	2,362 (4.70)	423 (17.91)	Ref	0	0	0	Little/no change
HiInc, HiEd, Swe, 65-84 years, W	152 (0.63)	42 (27.63)	1.21 (0.88-1.66)	631 (1.43)	124 (19.65)	0.92 (0.76-1.12)	1,479 (2.94)	225 (15.21)	0.85 (0.73-0.99)	-23.60	-8.11	-29.80 *	Pers dec
HiInc, HiEd, Swe, 45-64 years, M	1,551 (6.39)	294 (18.96)	0.83 (0.67-1.03)	2,803 (6.35)	567 (20.23)	0.95 (0.84-1.08)	3,364 (6.70)	580 (17.24)	0.96 (0.86-1.08)	14.64	1.17	15.98	Pers inc
HiInc, HiEd, Swe, 45-64 years, W	1,284 (5.29)	239 (18.61)	0.82 (0.66-1.01)	2,876 (6.51)	470 (16.34)	0.77 (0.67-0.88)	4,304 (8.57)	605 (14.06)	0.78 (0.70-0.88)	-5.69	2.10	-3.71	Del inc
HiInc, HiEd, Swe, 24-44 years, M	1,198 (4.94)	230 (19.20)	0.84 (0.68-1.05)	2,379 (5.39)	492 (20.68)	0.97 (0.85-1.11)	3,093 (6.16)	675 (21.82)	1.22 (1.09-1.36)	15.72	25.25*	44.94*	Pers inc
HiInc, HiEd, Swe, 24-44 years, W	891 (3.67)	141 (15.82)	0.69 (0.55-0.88)	2,127 (4.82)	347 (16.31)	0.77 (0.67-0.89)	3,116 (6.20)	467 (14.99)	0.84 (0.74-0.94)	10.74	9.04	20.76	Pers inc
HiInc, HiEd, Im, 65-84 years, M	24 (0.10)	5 (20.83)	0.91 (0.41-2.03)	135 (0.31)	31 (22.96)	1.08 (0.78-1.50)	225 (0.45)	44 (19.56)	1.09 (0.83-1.44)	18.40	1.08	19.69	Pers inc
HiInc, HiEd, Im, 65-84 years, W	13 (0.05)	1 (7.69)	0.34 (0.05-2.23)	83 (0.19)	15 (18.07)	0.85 (0.53-1.36)	170 (0.34)	28 (16.47)	0.92 (0.65-1.30)	152.38	8.18	173.01	Pers inc
HiInc, HiEd, Im, 45-64 years, M	83 (0.34)	27 (32.53)	1.42 (0.99-2.04)	427 (0.96)	129 (30.21)	1.42 (1.19-1.70)	537 (1.07)	139 (25.88)	1.45 (1.22-1.71)	-0.24	1.70	1.46	Del inc
HiInc, HiEd, Im, 45-64 years, W	64 (0.26)	19 (29.69)	1.3 (0.85-1.98)	486 (1.10)	127 (26.13)	1.23 (1.02-1.48)	657 (1.31)	144 (21.92)	1.22 (1.03-1.45)	-5.44	-0.44	-5.86	Little/no change
HiInc, HiEd, Im, 24-44 years, M	42 (0.17)	14 (33.33)	1.46 (0.92-2.33)	316 (0.72)	91 (28.80)	1.35 (1.11-1.66)	386 (0.77)	107 (27.72)	1.55 (1.29-1.86)	-7.19	14.26	6.04	Del inc

HiInc, HiEd, Im, 24-44 years, W	47 (0.19)	11 (23.40)	1.02 (0.59-1.77)	320 (0.72)	68 (21.25)	1 (0.79-1.27)	432 (0.86)	100 (23.15)	1.29 (1.07-1.57)	-2.46	29.30	26.11	Del inc
HiInc, LoEd, Swe, 65-84 years, M	219 (0.90)	52 (23.74)	1.04 (0.77-1.40)	830 (1.88)	215 (25.90)	1.22 (1.04-1.42)	1373 (2.73)	346 (25.20)	1.41 (1.24-1.60)	17.19	15.47	35.33	Pers inc
HiInc, LoEd, Swe, 65-84 years, W	112 (0.46)	29 (25.89)	1.13 (0.79-1.63)	420 (0.95)	91 (21.67)	1.02 (0.83-1.26)	818 (1.63)	171 (20.90)	1.17 (1.00-1.37)	-10.11	14.52	2.94	Del inc
HiInc, LoEd, Swe, 45-64 years, M	1,098 (4.53)	296 (26.96)	1.18 (0.96-1.45)	2,350 (5.32)	644 (27.40)	1.29 (1.14-1.46)	2902 (5.78)	784 (27.02)	1.51 (1.36-1.68)	9.20	17.01	27.78*	Pers inc
HiInc, LoEd, Swe, 45-64 years, W	480 (1.98)	96 (20.00)	0.88 (0.68-1.13)	1,306 (2.96)	259 (19.83)	0.93 (0.80-1.09)	1886 (3.75)	354 (18.77)	1.05 (0.92-1.19)	6.52	12.34	19.66	Pers inc
HiInc, LoEd, Swe, 24-44 years, M	612 (2.52)	168 (27.45)	1.2 (0.96-1.51)	796 (1.80)	251 (31.53)	1.48 (1.28-1.72)	454 (0.90)	134 (29.52)	1.65 (1.40-1.95)	23.40	11.10	37.10	Pers inc
HiInc, LoEd, Swe, 24-44 years, W	259 (1.07)	88 (33.98)	1.49 (1.16-1.91)	340 (0.77)	61 (17.94)	0.84 (0.66-1.08)	196 (0.39)	50 (25.51)	1.42 (1.10-1.84)	-43.28*	68.77*	-4.27	Del inc
HiInc, LoEd, Im, 65-84 years, M	9 (0.04)	3 (33.33)	1.46 (0.57-3.75)	68 (0.15)	28 (41.18)	1.94 (1.43-2.62)	93 (0.19)	28 (30.11)	1.68 (1.22-2.32)	32.70	-13.21	15.17	Rebound
HiInc, LoEd, Im, 65-84 years, W	2(0.01)	0(0)	Not estimated	46 (0.10)	16 (34.78)	1.64 (1.09-2.46)	71 (0.14)	12 (16.90)	0.94 (0.56-1.59)	-	-	-	Not estimated
HiInc, LoEd, Im, 45-64 years, M	42 (0.17)	18 (42.86)	1.88 (1.26-2.79)	254 (0.58)	107 (42.13)	1.98 (1.66-2.37)	253 (0.50)	99 (39.13)	2.19 (1.83-2.61)	5.59	10.26	16.42	Pers inc
HiInc, LoEd, Im, 45-64 years, W	26 (0.11)	7 (26.92)	1.18 (0.61-2.28)	172 (0.39)	40 (23.26)	1.09 (0.82-1.46)	197 (0.39)	56 (28.43)	1.59 (1.25-2.01)	-7.21	45.09	34.63	Del inc
HiInc, LoEd, Im, 24-44 years, M	20 (0.08)	8 (40.00)	1.75 (0.99-3.09)	94 (0.21)	32 (34.04)	1.6 (1.19-2.16)	76 (0.15)	32 (42.11)	2.35 (1.78-3.10)	-8.58	46.81	34.22	Del inc
HiInc, LoEd, Im, 24-44 years, W	8 (0.03)	1 (12.50)	0.55 (0.09-3.46)	58 (0.13)	23 (39.66)	1.87 (1.34-2.61)	47 (0.09)	17 (36.17)	2.02 (1.37-2.98)	240.79	8.27	268.96	Pers inc
LoInc, HiEd, Swe, 65-84 years, M	328 (1.35)	104 (31.71)	1.39 (1.09-1.77)	845 (1.91)	259 (30.65)	1.44 (1.25-1.67)	1,200 (2.39)	374 (31.17)	1.74 (1.54-1.96)	3.84	20.69	25.33	Pers inc
LoInc, HiEd, Swe, 65-84 years, W	356 (1.47)	84 (23.60)	1.03 (0.79-1.34)	1,051 (2.38)	239 (22.74)	1.07 (0.92-1.25)	1,675 (3.33)	339 (20.24)	1.13 (0.99-1.28)	3.53	5.64	9.37	Pers inc
LoInc, HiEd, Swe, 45-64 years, M	551 (2.27)	191 (34.66)	1.52 (1.22-1.89)	599 (1.36)	190 (31.72)	1.49 (1.27-1.75)	464 (0.92)	158 (34.05)	1.9 (1.63-2.22)	-1.70	27.42*	25.25	Del inc

LoInc, HiEd, Swe, 45-64 years, W	1,196 (4.93)	311 (26.00)	1.14 (0.93-1.40)	1,559 (3.53)	353 (22.74)	1.07 (0.93-1.22)	1,086 (2.16)	250 (23.02)	1.29 (1.12-1.48)	-6.46	20.68	12.88	Del inc
LoInc, HiEd, Swe, 24-44 years, M	1,350 (5.56)	441 (32.67)	1.43 (1.17-1.75)	1,377 (3.12)	399 (28.98)	1.36 (1.19-1.56)	1,279 (2.55)	367 (28.69)	1.6 (1.42-1.81)	-4.71	17.54	12.00	Del inc
LoInc, HiEd, Swe, 24-44 years, W	2,813 (11.59)	600 (21.33)	0.93 (0.77-1.14)	3,453 (7.82)	685 (19.84)	0.93 (0.82-1.06)	2,813 (5.60)	567 (20.16)	1.13 (1.00-1.26)	-0.09	20.60*	20.49	Del inc
LoInc, HiEd, Im, 65-84 years, M	26 (0.11)	9 (34.62)	1.52 (0.87-2.65)	166 (0.38)	69 (41.57)	1.96 (1.59-2.41)	235 (0.47)	81 (34.47)	1.92 (1.58-2.34)	28.99	-1.57	26.96	Rebound
LoInc, HiEd, Im, 65-84 years, W	21 (0.09)	5 (23.81)	1.04 (0.47-2.29)	205 (0.46)	55 (26.83)	1.26 (0.98-1.62)	237 (0.47)	77 (32.49)	1.81 (1.48-2.22)	21.05	43.74*	73.99	Pers inc
LoInc, HiEd, Im, 45-64 years, M	69 (0.28)	34 (49.28)	2.16 (1.59-2.92)	292 (0.66)	147 (50.34)	2.37 (2.03-2.77)	227 (0.45)	93 (40.97)	2.29 (1.91-2.73)	9.75	-3.40	6.01	Rebound
LoInc, HiEd, Im, 45-64 years, W	101 (0.42)	42 (41.58)	1.82 (1.35-2.45)	422 (0.96)	155 (36.73)	1.73 (1.47-2.03)	373 (0.74)	136 (36.46)	2.04 (1.74-2.39)	-5.12	17.83	11.80	Del inc
LoInc, HiEd, Im, 24-44 years, M	82 (0.34)	36 (43.90)	1.92 (1.42-2.61)	372 (0.84)	141 (37.90)	1.78 (1.51-2.11)	312 (0.62)	98 (31.41)	1.75 (1.46-2.11)	-7.26	-1.64	-8.77	Pers dec
LoInc, HiEd, Im, 24-44 years, W	180 (0.74)	53 (29.34)	1.29 (0.96-1.73)	770 (1.74)	239 (31.04)	1.46 (1.26-1.69)	595 (1.18)	181 (30.42)	1.7 (1.46-1.97)	13.24	16.33	31.73	Pers inc
LoInc, LoEd, Swe, 65-84 years, M	1,027 (4.23)	355 (34.57)	1.51 (1.24-1.85)	2,117 (4.80)	711 (33.59)	1.58 (1.40-1.78)	2,751 (5.48)	936 (34.02)	1.9 (1.72-2.10)	4.37	20.25*	25.51*	Pers inc
LoInc, LoEd, Swe, 65-84 years, W	1,421 (5.86)	453 (31.88)	1.4 (1.14-1.70)	3,347 (7.58)	901 (26.92)	1.27 (1.12-1.43)	4,073 (8.11)	1,076 (26.42)	1.48 (1.33-1.63)	-9.29	16.48	5.66	Del inc
LoInc, LoEd, Swe, 45-64 years, M	1,328 (5.47)	502 (37.80)	1.66 (1.36-2.02)	1,265 (2.87)	499 (39.45)	1.86 (1.64-2.10)	817 (1.63)	362 (44.31)	2.47 (2.20-2.78)	12.10	33.33*	49.46*	Pers inc
LoInc, LoEd, Swe, 45-64 years, W	2,440 (10.06)	715 (29.30)	1.28 (1.06-1.56)	2,595 (5.88)	758 (29.21)	1.37 (1.22-1.55)	1,546 (3.08)	501 (32.41)	1.81 (1.62-2.02)	7.08	31.68*	41.01*	Pers inc
LoInc, LoEd, Swe, 24-44 years, M	832(3.4 3)	320 (38.46)	1.68 (1.37-2.06)	461 (1.04)	193 (41.87)	1.97 (1.69-2.29)	233 (0.46)	99 (42.49)	2.37 (2.00-2.82)	16.93	20.46	40.86*	Pers inc
LoInc, LoEd, Swe, 24-44 years, W	1,089 (4.49)	357 (32.78)	1.44 (1.17-1.76)	769 (1.74)	231 (30.04)	1.41 (1.22-1.64)	367 (0.73)	145 (39.51)	2.21 (1.89-2.57)	-1.57	56.12*	53.67*	Del inc
LoInc, LoEd, Im, 65-84 years, M	32 (0.13)	16 (50.00)	2.19 (1.48-3.24)	245 (0.55)	108 (44.08)	2.07 (1.74-2.47)	270 (0.54)	113 (41.85)	2.34 (1.98-2.76)	-5.29	12.69	6.73	Del inc

LoInc, LoEd, Im, 65-84 years, W	67 (0.28)	23 (34.33)	1.5 (1.03-2.20)	423 (0.96)	162 (38.30)	1.8 (1.53-2.11)	427 (0.85)	133 (31.15)	1.74 (1.47-2.05)	19.84	-3.46	15.69	Rebound
LoInc, LoEd, Im, 45-64 years, M	87 (0.36)	49 (56.32)	2.47 (1.90-3.20)	288 (0.65)	156 (54.17)	2.55 (2.19-2.96)	194 (0.39)	101 (52.06)	2.91 (2.48-3.41)	3.31	14.08	17.86	Pers inc
LoInc, LoEd, Im, 45-64 years, W	139 (0.57)	55 (39.57)	1.73 (1.31-2.28)	474 (1.07)	199 (41.98)	1.98 (1.70-2.29)	288 (0.57)	115 (39.93)	2.23 (1.89-2.63)	13.98	12.89	28.67	Pers inc
LoInc, LoEd, Im, 24-44 years, M	45 (0.19)	25 (55.56)	2.43 (1.77-3.35)	170 (0.39)	87 (51.18)	2.41 (2.01-2.88)	116 (0.23)	52 (44.83)	2.5 (2.01-3.12)	-1.04	3.97	2.88	Del inc
LoInc, LoEd, Im, 24-44 years, W	66 (0.27)	30 (45.45)	1.99 (1.44-2.75)	277 (0.63)	127 (45.85)	2.16 (1.83-2.55)	170 (0.34)	71 (41.76)	2.33 (1.91-2.84)	8.35	8.12	17.16	Pers inc

HiInc = high income, HiEd = high education, LoInc = low income, LoEd = low education, Swe = Swedish, Im = immigrant, M= man, W= woman

*Significant comparison tested by interaction effects (p<0.05)

Per inc = persistent increased inequities, Per dec = persistent decreased inequities, Rebound = rebounding inequities, Del inc = delayed increased inequities.
